# Physical activity, screen time and dietary behaviours in New Zealand adolescents prior to and following the onset of the COVID-19 pandemic

**DOI:** 10.1186/s12889-024-17688-7

**Published:** 2024-01-16

**Authors:** Sandra Mandic, Asaduzzaman Khan, Enrique García Bengoechea, Kirsten J. Coppell, John C. Spence, Melody Smith

**Affiliations:** 1 AGILE Research Ltd, Wellington, New Zealand; 2 School of Sport and Recreation, Faculty of Health and Environmental Sciences, Auckland University of Technology, Private Bag 92006, Auckland, 1142 New Zealand; 3 Centre for Sustainability, University of Otago, Dunedin, New Zealand; 4 School of Health and Rehabilitation Sciences, Faculty of Health and Behavioural Sciences, University of Queensland, Brisbane, Australia; 5 Physical Activity for Health Research Cluster, Health Research Institute, Department of Physical Education and Sport Sciences, University of Limerick, Limerick, Ireland; 6 Research and Innovation Unit, Sport Ireland, Dublin, Ireland; 7 Department of Medicine, University of Otago, Wellington, New Zealand; 8 Nelson Marlborough Institute of Technology, Nelson, New Zealand; 9 Faculty of Kinesiology, Sport, and Recreation, University of Alberta, Edmonton, Canada; 10 School of Nursing, Faculty of Medical and Health Sciences, The University of Auckland, Auckland, New Zealand

**Keywords:** Physical activity, Sedentary behaviour, Diet, Lifestyle, Youth

## Abstract

**Background:**

Insufficient physical activity, high screen time, and unhealthy dietary patterns among adolescents may have worsened during the pandemic, but data are lacking. This study compared physical activity, screen time and fruit and vegetable intake in adolescents from Dunedin, New Zealand, 5–6 years before (Study 1) and during (Study 2) the COVID-19 pandemic.

**Methods:**

Adolescents completed an online survey as part of the Built Environment and Active Transport to School (BEATS) studies in 2014/2015 (Study 1; *n* = 1,266; age: 15.3 ± 1.4 years; 54.6% female) and 2021/2022 (Study 2; *n* = 819; age: 15.2 ± 1.4 years; 47.4% female). The proportion of adolescents meeting guidelines for physical activity (≥ 60 min/day of moderate-to-vigorous physical activity), outside school screen time (≤ 2 h/day) and fruit and vegetable intake (> 1 serving/day for both fruit and vegetables) was calculated. Data were analysed using multivariable linear and logistic regression modelling.

**Results:**

Few adolescents met recommended health behaviour guidelines. Compared to Study 1, significantly greater proportions of adolescents at Study 2 met guidelines for physical activity (16.7% vs. 23.1%; *p* < 0.001) and outside school screen time (13.3% vs. 18.3%; *p* < 0.001) while fruit and vegetable intake was not different (29.6% vs. 27.0%; *p* = 0.322). Compared to Study 1, average outside school screen time at Study 2 was lower on both weekdays (5.0 ± 2.9 vs. 4.6 ± 2.9; *p* < 0.001) and weekend days (6.9 ± 3.5 vs. 6.1 ± 3.6 h/day; *p* < 0.001). Reported frequency of consuming sweets was higher and soft drinks lower at Study 2 versus Study 1.

**Conclusions:**

Despite observed higher levels of physical activity and lower levels of outside school screen time during the pandemic compared to the pre-pandemic levels, few adolescents met health behaviour guidelines at both time points. Therefore, comprehensive health promotion that aims to improve physical activity levels, screen time and dietary patterns for adolescents is still necessary to prevent chronic health conditions adulthood.

**Supplementary Information:**

The online version contains supplementary material available at 10.1186/s12889-024-17688-7.

## Background

Patterns of unhealthy lifestyle behaviours in adulthood often begin during childhood and adolescence [1] and increase the risk of obesity and many non-communicable diseases like type 2 diabetes, hypertension and non-alcoholic fatty liver disease [2]. Insufficient physical activity (PA) [3], high screen time (ST) [3], and unhealthy dietary patterns [4, 5] were reported in adolescents from developed countries prior to the COVID-19 pandemic. Globally, four out of five adolescents are not sufficiently physically active and little progress has been accomplished since the beginning of the century, particularly for girls [6, 7].

In New Zealand, the most recent Physical Activity Report Card showed low and inequitable levels of meeting PA and sedentary time guidelines in adolescents (age 15–18 years) [8]. Less than half (47%) of 15 to 18-year olds met the PA guidelines, compared with 60% of 5 to 11-year olds and 62% of 12- to 15-year olds. Similarly, only 12% of 15- to 18-year olds met the sedentary time guidelines, compared with 61% of 5- to 11-year olds and 36% of 12- to 15-year olds.

Globally adolescents’ dietary patterns have deteriorated over time [9]. While there is inter-country variation, the overall median prevalence rates of insufficient fruit and vegetable intakes (defined as less than two servings of fruits and less than three servings of vegetables per day) amongst 11- to 17-year-old adolescents were 68% and 79%, respectively, for the 2013–2017 period. During the same period, 37% of 11- to 17-year-old adolescents regularly consumed soft drinks and 33% regularly consumed fast foods high in fat, sugar and salt [9]. These food habits varied widely between countries, for example, median insufficient fruit intake ranged from 27% in Mauritas to 93% in Greenland and median soft drink consumption was 2% in Finland compared with 85% in Qatar. Dietary data specifically for New Zealand adolescents are limited but consistent with international data. A 2012 national study observed that 83% of adolescents consumed less than 5 servings of fruit and vegetables daily [10].

In addition, lifestyle-related risk factors tend to cluster and co-occur in adolescents, exacerbating negative health outcomes [11–14]. Clustering of PA, sedentary time and dietary behaviours in this age group has been previously reported in European [15–17], American [18], Canadian [19, 20], Australian [21] and New Zealand adolescents [22] as well as globally [9, 23]. This clustering of lifestyle risk factors in adolescents has implications for intervention approaches aimed at improving lifestyle behaviours in adolescents [24].

Emerging international evidence suggests these lifestyle behaviours worsened during the pandemic. Systematic reviews reveal that screen time increased by an average of 110 min per day [25] and moderate-to-vigorous physical activity (MVPA) decreased by an average of 17 min [26] per day in comparison to pre-pandemic levels. For example, the proportion of U.S. adolescents meeting PA guidelines of at least 60 min of MVPA per day decreased from 16.1% to 8.9% with the onset of the pandemic [27, 28]. However, there are no data for many countries, including New Zealand. In New Zealand, PA in young people was relatively stable from 2017 to 2019, but in 2021 all key physical activity indicators (weekly participation, meeting PA guidelines, and average PA hours per week) dropped significantly for those aged 15–17 years [29]. The 2020 Growing Up in New Zealand Life During Lockdown study with 2421 children aged 10–11 years [30] showed a doubling of screen time since 2018, however it is unclear whether this was due to lockdowns, and whether this level of screen time has been sustained.

The New Zealand response to COVID-19 was rapid and amongst the most stringent internationally [31]. The first case was reported on 28 February 2020, and within three weeks the country adopted a four-tier alert system, with levels 3 and 4 signaling ‘lockdown’. At these levels, country borders, non-essential services, and playgrounds were closed; essential travel only was permitted (e.g., for urgent medical treatment); and households were strongly encouraged to avoid physical contact with others. Schools were closed at level 4 and re-opened for children of essential workers only at level 3. Alert level 2 signaled re-opening of schools for all children and young people and all businesses were able to open, but country borders remained closed. A nationwide lockdown occurred from 23 March to 13 May 2020, and with the exception of Auckland (which had multiple lockdowns), the country remained at levels 1 or 2 for over a year, but went back into Alert levels 3 and 4 from 31 August – 7 September 2021. A recent article [32] provides a full overview of the COVID-19 response in relation to children and young people in New Zealand.

With specific regard to youth, lockdowns in New Zealand removed numerous opportunities to participate in PA through travel to and from school, activity at school (e.g., during breaks but also moving between classes), extra-curricular activities and participation in organised sports, and social activities with family and friends. Learning was delivered virtually, and relationships were also maintained through virtual means, likely resulting in considerably higher levels of screen time than pre-COVID. Conversely, opportunities to be more physically active than usual arose, for example through significant reduction of car travel allowing for safe and enjoyable active travel in the neighbourhood [32].

Overall, the evidence suggests that the COVID-19 pandemic negatively impacted adolescents’ health behaviours. However, heterogeneity in findings exists, studies have been limited in understanding a range of health behaviours (and clustering of these), and differences across countries have been observed. It is likely that New Zealand’s unique combination of stringent lockdowns and prolonged periods with few restrictions during the COVID-19 pandemic may have impacted adolescents’ health behaviours in different ways than in other countries. There is a clear need for more detailed evidence on changes over time in a range of health behaviours at the local level. Detailed evidence on changes during the COVID-19 pandemic at the local level is important because it may inform the development of policies and actions needed to counter the negative impacts that similar events and circumstances may have on health behaviours of adolescents. This study compared PA, outside school ST and dietary behaviours in New Zealand adolescents five to six years before (study 1 (S1)) and one to two years following (study 2 (S2)) the onset of the COVID-19 pandemic on 28 February 2020.

## Methods

### Setting

This research analysed data from a sample of adolescents in the city of Dunedin (total population: ≈120,000), Aotearoa New Zealand who participated in two studies: the Built Environment and Active Transport to School (BEATS) Study [33] in 2014–2015 (1,780 adolescents; study 1 (S1)) or BEATS Natural Experiment [34] in 2020–2022 (1,828 adolescents; study 2 (S2)). For both studies, data were collected throughout the school year which included fall, winter and spring seasons. Dunedin is a mid-sized city on the lower South Island of New Zealand. All 12 secondary schools in the city participated in both BEATS studies.

### Participants

**Fig. 1:**
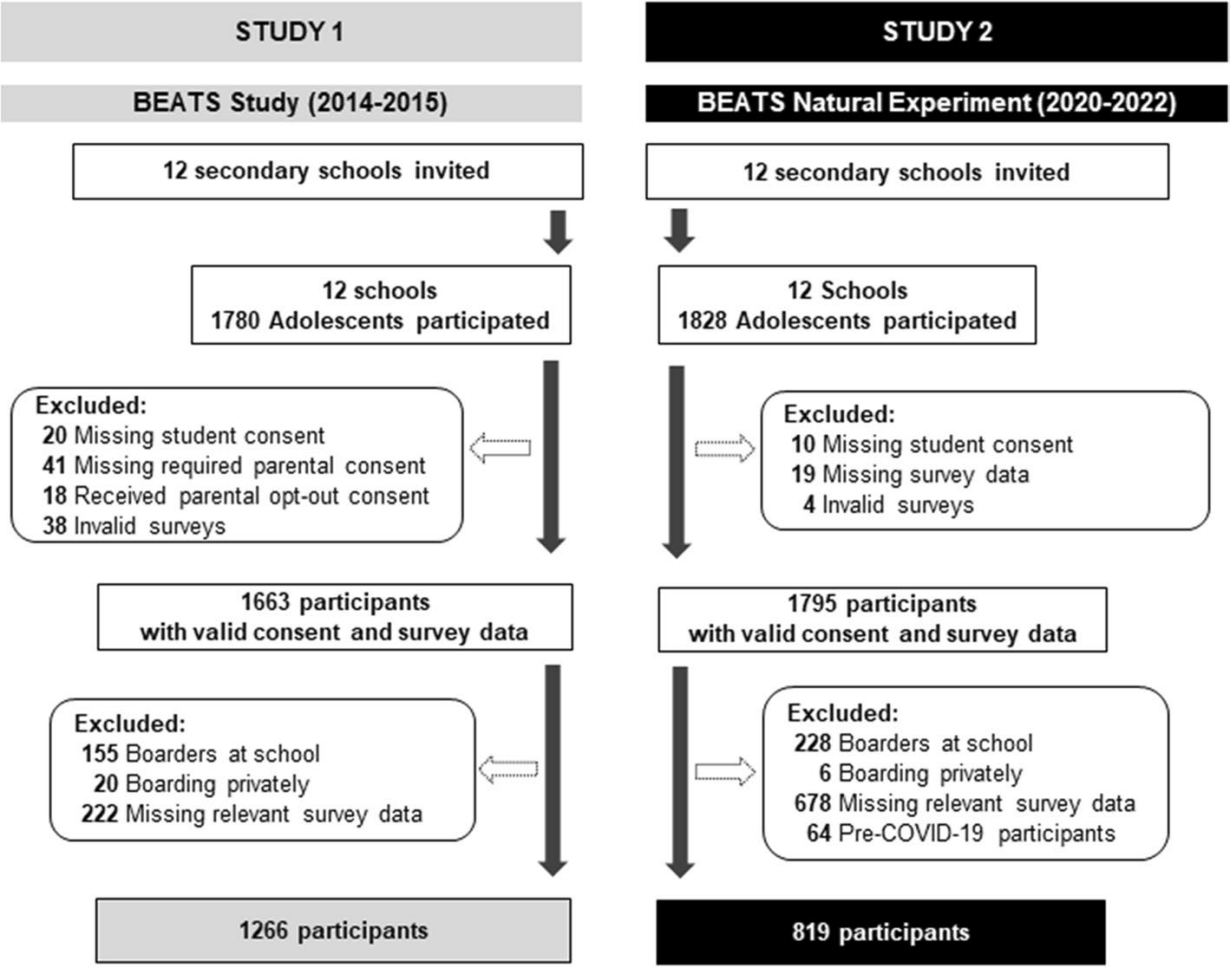
Flow chart of participant recruitment and selection

For both studies, adolescents (age: 13 to 18 years; school years 9 to 13) were recruited through their school, as previously described in separate study protocols [33, 34]. Schools delivered study-related information to invited adolescents. If interested, adolescents signed written consent prior to participation. In the BEATS Study, parental opt-in or opt-out consent was used for adolescents under 16 years of age, based on schools’ preference. Parental consent was not required in the BEATS Natural Experiment. Both projects received ethical approval from the University of Otago Human Ethics Committee (BEATS: 13/203; BEATS Natural Experiment: 17/188) and Auckland University of Technology Ethics Committee (BEATS Natural Experiment: 21/314). Figure 1 presents a flow chart of participant recruitment, exclusions and final selection of participants for this data analysis. Data from 1,266 adolescents were available in 2014/2015 (S1) and 819 adolescents in 2021/2022 (S2). Adolescents who completed the survey one to three weeks prior to the first country-wide COVID-19 related lockdown in March 2020 were excluded from the analysis, which also removed one school from the S2 sample. Therefore, the S2 sample includes adolescents surveyed during the 14-month period between May 2021 and June 2022 (14 to 25 months after the onset of the COVID-19 pandemic in New Zealand).

### Survey

In both studies, adolescents completed a 30- to 40-minute online survey during a school period, under the supervision of research staff. Participants self-reported their sociodemographic characteristics, home address, selected family resources, health behaviours (PA, outside school ST (TV/computer/video games) and dietary behaviours) and perceived health.

### Sociodemographic characteristics

Participants self-reported their date of birth, gender, and ethnicity. Age at survey was calculated from date of birth. Adolescents were categorized into five ethnic groups using prioritized ethnicity for New Zealand: ‘Māori’, ‘Pacific’, ‘Asian’, ‘New Zealand European’ and ‘Other’ [35] and subsequently also re-categorised into three ethnic groups for multivariable analysis: ‘Māori’, ‘New Zealand European’ and ‘Other’. In the BEATS Study, area level deprivation was calculated using the New Zealand Index of Deprivation for 2013 (NZDep2013) [36] and in the BEATS Natural Experiment the updated NZDep2018 was used [37, 38], as described previously [33, 34]. The NZDep is an area-level measure of deprivation, calculated at the meshblock (2013) or Statistical Area Unit (SA1) level, using Census data for nine variables which have remained relatively constant over time. Meshblocks are the smallest geographical area defined by Statistics New Zealand, comprising around 60–110 residents; SA1s are larger (generally 100–200 residents but can have up to 500). The NZDep index for each individual was calculated in GIS using participant address data to allocate them to a meshblock (2013) or SA1 (2018) [37].

### Physical activity

MVPA was assessed using the question “Over the past 7 days, on how many days were you physically active for a total of at least 60 minutes per day?” [39]. This question was preceded by a concise explanation of the intended meaning of ‘physical activity’. Adolescents were categorised as meeting PA guidelines if they self-reported ≥ 60 min of MVPA for 7 days [40–42] according to the PA guidelines prior to the new PA guidelines for children and adolescents released by the World Health Organization in 2020 [43].

### Sports participation

Adolescents reported sport participation using a question “Are you involved in any sport or do you belong to any sports teams?” with separate questions for sports “at school” and “outside of school”, using ‘yes’ or ‘no’ response categories.

### Outside school screen time

Outside school ST was assessed using the questions “About how many hours a day do you usually watch television / play games on a computer or games console / use a computer for chatting on-line, internet, emailing, homework etc.) in your free time?”. Separate questions were asked for each activity and for weekdays and weekend days [39]. Adolescents selected one of 10 response categories (ranging from ‘none at all’, ‘rarely’, ‘about half an hour a day or less’, ‘about 1 hour a day’ to ‘about 7 or more hours per day’). Total weekly outside school ST was calculated by combining weekday and weekend estimates for TV watching, playing computer games and internet use and included homework, as described previously [22]. To calculate average screen time, collected categorical screen time data for each survey item were recoded as follows: ‘none at all’ = ‘0 hours/day’; ‘rarely’ = ‘0.1 hours/day’; ‘about half an hour a day or less’ = ‘0.5 hours/day’; ‘about 1 hour per day’ = ‘1 hour/day’; ‘about 2 hours per day’ = ‘2 hours/day’; ‘about 3 hours per day’ = ‘3 hours/day’; ‘about 4 hours per day’ = ‘4 hours/day’; ‘about 5 hours per day’ = ‘5 hours/day’; ‘about 6 hours per day’ = ‘6 hours/day’; and ‘about 7 or more hours per day’ = ‘7 hours/day’. A threshold value of ≤ 2 hours of outside school ST per day was used to identify adolescents meeting ST guidelines [41, 42].

### Dietary habits

Weekly intake of fruit and vegetables, sweets, soft drinks and fast foods was assessed using the question “How many times a week do you usually eat…?” [39]. Participants selected one of seven response categories: “never”; “less than once a week”; “once a week”; “2–4 days a week”; “5–6 days a week”; “once a day, every day” and “every day, more than once”. Since these data were not normally distributed, participants’ responses were subsequently recoded into 3-category variables for weekly consumption in each food category: ‘once a week or less’, ‘2 to 4 times per week’ or ‘5 or more times per week’ for descriptive analysis of dietary patterns. Participants who self-reported consuming both fruit and vegetables “every day, more than once” were classified as meeting fruit and vegetable recommendations, which provided an indication of whether dietary recommendations were met or not [42].

The number of health behaviour guidelines met by each adolescent was calculated as a sum of meeting individual guidelines for PA (≥ 60 min of MVPA/day), outside school ST (≤ 2 h/day) and fruit and vegetable intake (> 1/day each for both fruit and vegetables) and ranged from zero to three, as described previously [22].

### Perceived health

Self-perceived health was assessed using a single question: “In general, how would you say your health is?” [39]. Response categories were ‘excellent’, ‘very good’, ‘good’, ‘fair’ and ‘poor’.

### Data analysis

For descriptive statistics, categorical variables are reported as frequency (percentage) whereas continuous variables are reported as mean ± SD. Differences in outcome variables of interest between the study 1 and study 2 were examined using multivariable regression modelling, adjusted by age, gender (‘female’ or ‘male’), ethnicity (‘Māori’, ‘New Zealand European’ or ‘Other’) and neighbourhood deprivation score. Logistic regression modelling was used for examining binary outcomes (e.g., meeting PA guidelines, meeting ST guidelines), multinomial logistic regression modelling was used for examining polychotomous outcomes (e.g., transport to school, consumption of sweets) and linear regression modelling was used for examining continuous outcomes (e.g., average weekday or weekend day ST). P-level of less than 0.05 was considered statistically significant. Data were analyzed using SPSS 27.0 and StataSE 17.

## Results

### Sociodemographic characteristics

This analysis was based on data from 2,085 adolescents (S1: 1,264 adolescents from 12 schools; S2: 819 adolescents from 11 schools) (Table 1). Gender, ethnicity, neighbourhood level deprivation and self-reported health differed between S1 and S2 participants (Table 1). The proportion of males, adolescents of Māori and ‘other’ ethnic groups was higher at S2 compared to S1, while the proportion of New Zealand European adolescents was higher at S1 compared to S2. Compared to S1, at S2 on average adolescents lived in households with more vehicles, more desktop and laptop computers and fewer televisions and game consoles. Differences in neighbourhood level deprivation and self-reported health were also observed between the samples appearing to indicate a slight deterioration in the latter case.

**Table 1:** Sociodemographic and family characteristics of adolescent participants

	Study 1 (2014/15)	Study 2 (2021/22)	*p*-value
	*n*=1266	*n*=819	
Age (years)	15.3 ± 1.4	15.2 ± 1.4	0.171
Gender [n(%)]			
Male	575 (45.4%)	409 (49.9%)	
Female	691 (54.6%)	388 (47.4%)	
Gender diverse^a^	N/A	22 (2.7%)	<0.001
Ethnicity [n(%)]			
New Zealand European	931 (73.5%)	538 (65.7%)	
Māori	142 (11.2%)	120 (14.7%)	
Pacific	47 (3.7%)	25 (3.1%)	
Asian	80 (6.3%)	36 (4.4%)	
Other	60 (4.7%)	100 (12.2%)	<0.001
Neighbourhood deprivation score (%)	(*n*=1240)	(*n*=813)	
1 (least deprived)	385 (31.0%)	252 (31.0%)	
2	302 (24.0%)	226 (27.8%)	
3	257 (20.7%)	124 (15.3%)	
4	181 (14.6%)	134 (16.5%)	
5 (most deprived)	155 (9.3%)	77 (9.5%)	0.023
Self-perceived health			
Excellent	224 (17.7%)	134 (16.4%)	
Very good	559 (44.2%)	325 (39.7%)	
Good	387 (30.6%)	275 (33.6%)	
Fair	82 (6.5%)	77 (9.4%)	
Poor	14 (1.1%)	8 (1.0%)	0.042
**Family structure**			
Number of people living at home (n)	4.2 ± 1.3	4.3 ± 1.2	0.427
Number of adults at home (n)	1.9 ± 0.6	2.0 ± 0.5	<0.001
Number of siblings (n)	2.2 ± 1.6	1.9 ± 1.3	0.003
**Resources at home**			
Number of bikes available to use to get to school (%)			
None	24.2%	23.3%	
One	20.1%	18.6%	
Two or more	55.7%	58.1%	0.528
Number of vehicles at home (%)			
None	3.5%	0.9%	
One	26.5%	19.0%	
Two or more	70.1%	80.1%	<0.001
Number of televisions (n)	2.4 ± 1.1	2.2 ± 1.1	<0.001
Number of desktop computers (n)	0.9 ± 0.9	1.1 ± 1.2	0.026
Number of laptops (n)	2.0 ± 1.2	2.8 ± 1.1	<0.001
Number of game consoles (n)	1.4 ± 1.1	1.3 ± 1.2	0.003

^a^Note: Gender diverse response category was used only in Study 2. Those data were not collected in Study 1.

### Meeting health behaviours guidelines

**Fig. 2:**
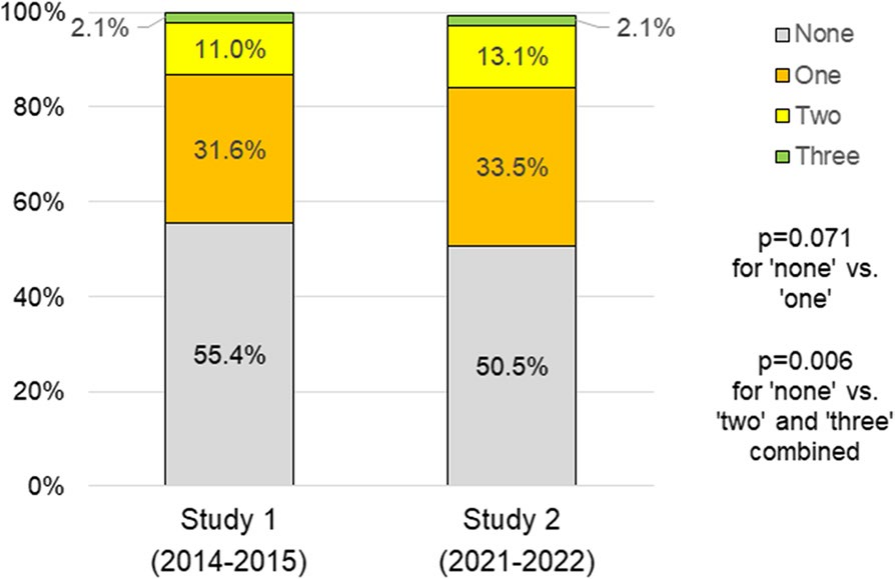
Proportion of adolescents meeting guidelines for physical activity, screen time and fruit and vegetable intake

In both samples, few adolescents met health behaviour guidelines (Table 2). Compared to S1, a greater proportion of S2 adolescents met guidelines for PA (16.7% vs. 23.1%; *p* < 0.001) and ST (13.3% vs. 18.3%; *p* < 0.001) while no significant difference was observed for fruit and vegetable intake (29.6% vs. 27.0%; *p* = 0.322) (Table 2) and all three health behaviours combined (Fig. 2). Average outside school ST was lower on both weekdays (5.0 ± 2.9 vs. 4.6 ± 2.9; *p* < 0.001) and weekend days (6.9 ± 3.5 vs. 6.1 ± 3.6 h/day; *p* < 0.001) in S2 compared to S1. A higher weekly frequency of consuming sweets and a lower frequency of consuming soft drinks and fast food were found in S2 versus S1 (Table 2).

**Table 2:** Comparison of health behaviours in adolescents in study 1 versus study 2, using multivariable modelling

	Study 1 (2014-2015)	Study 2 (2021-2022)	*p*-value^a^
	*n*=1266	*n*=819	
**Physical activity**			
Meeting PA guidelines (≥60 min MVPA/day) (%)	16.7%	23.1%	<0.001
MVPA 60+ min/day (days/week)	4.1 ± 2.1	4.5 ± 2.0	<0.001
Sport participation at school (%)	67.2%	65.1%	0.475
Sport participation outside school (%)	50.2%	48.1%	0.437
**Screen time**			
Meeting screen time guidelines (≤2 hrs/day) (%)	13.3%	18.3%	<0.001
Watching television (including videos and DVDs) (hrs/day)	2.0 ± 1.7	1.8 ± 1.7	0.001
Playing games on a computer or games console (hrs/day)	1.3 ± 1.8	1.5 ± 1.9	0.489
Using a computer for chatting on-line, internet, emailing, homework, etc. (hrs/day)	2.7 ± 2.1	2.1 ± 1.9	<0.001
Weekday average (hrs/day)	5.0 ± 2.9	4.6 ± 2.9	<0.001
Weekend average (hrs/day)	6.9 ± 3.5	6.1 ± 3.6	<0.001
Daily average screen time (hrs/day)	5.6 ± 3.0	5.0 ± 3.0	<0.001
**Dietary habits**			
Meeting fruit and vegetable guidelines (more than once a day for both fruit and vegetables) (%)	29.6%	27.0%	0.322
Fruit consumption			
Once a week or less (Ref.)	8.9%	10.6%	
2-4 times per week	16.7%	18.7%	0.844
5 or more times per week	74.3%	70.7%	0.400
Vegetable consumption			
Once a week or less (Ref.)	4.8%	5.4%	
2-4 times per week	12.2%	9.6%	0.099
5 or more times per week	82.9%	85.0%	0.709
Sweet (candies and chocolate) consumption			
Once a week or less (Ref.)	42.6%	33.3%	
2-4 times per week	35.5%	41.6%	<0.001
5 or more times per week	21.9%	25.0%	0.003
Soft drink consumption			
Once a week or less (Ref.)	56.6%	70.0%	
2-4 times per week	25.4%	18.3%	<0.001
5 or more times per week	17.9%	11.7%	<0.001
Fast food consumption			
Once a week or less (Ref.)	82.3%	85.3%	
2-4 times per week	12.6%	10.6%	0.037
5 or more times per week	5.1%	4.0%	0.089

^a^ *P*-values are based on multivariable regression modelling, adjusted for age, gender (female, male), ethnicity (New Zealand European, Māori, Other) and New Zealand index of deprivation (quintiles).

Compared to adolescents who did not meet any of the health behaviours guidelines for PA, ST or fruit and vegetable intake, the proportion of adolescents who met two or three guidelines were significantly higher at S2 compared to S1 (*p* = 0.006; Fig. 2), whereas the difference between adolescents meeting one and no guideline did not reach statistical significance (*p* = 0.071; Fig. 2).

## Discussion

Key findings of this study are: (1) few adolescents met health behaviour guidelines both before and following the onset of the COVID-19 pandemic; (2) compared to 5–6 years before the pandemic, a greater proportion of adolescents met guidelines for PA and ST following the onset of the pandemic while no significant difference was observed for fruit and vegetable intake; (3) average outside school ST following the onset of the COVID-19 pandemic was comparatively lower than before the pandemic on both weekdays and weekend days; and (4) compared to the pre-pandemic period, adolescents self-reported higher weekly intake of sweets and lower weekly intake of soft drinks and fast food following the onset of the pandemic.

Overall, low levels of PA were observed, with less than a quarter of adolescents meeting PA guidelines at both time points. Significantly more adolescents reported meeting PA guidelines during the COVID-19 pandemic, compared with 5–6 years prior. This is in contrast with previous international literature reviews which have generally reported reductions in PA during the COVID-19 pandemic [26, 44–47]. These reductions are estimated as being between 11 min and 91 min per day [44] thus having significant potential to negatively impact health. These reviews have also highlighted inequities in the negative impact of COVID-19 on PA, particularly among older youth, those residing in higher deprivation areas, and those with less opportunities to be active in their neighbourhoods. Conflicting findings have been reported for gender, with one review noting greater reductions for males [45] and others reporting greater reductions for females [44, 46, 47]. One study also highlighted that reductions in PA appeared more marked for those who previously participated in organised team sports [45]. In our study, we controlled for key socio-demographic factors, and asked about overall PA and sports participation both at school and outside school time, yielding useful and robust insights about changes in different activity behaviours of importance.

While the increase in PA in the current study may seem counter-intuitive, it is consistent with other New Zealand research. For example, a survey of children and young people during COVID-19 showed a majority reported being active in their neighbourhood more than usual during COVID-19 lockdowns [32]. The Active NZ survey showed significant reductions in organised sports activities during the COVID-19 pandemic compared with earlier survey waves, but an increase in informal sports activities, resulting in an overall increase in average time spent being physically active [29]. This is in line with findings from two recent reviews [44, 46] which noted that if increases in PA were observed, these were generally in relation to unstructured and outdoor activities.

Unexpectedly, we found no significant differences in sport participation either at school or outside school. It is likely the timing of the surveys impacted how adolescents responded to the survey questions. All BEATS Natural Experiment (S2) data collection occurred at alert levels 1 and 2, when adolescents were able to attend school and physical distancing restrictions were reduced. As such, participation in school sports may have returned to usual levels during this time. Had the surveys been undertaken while schools were closed and physical distancing requirements were in place (as has been the case in some studies including the Active NZ survey), school sports participation would be expected to be non-existent. It is also possible the wording of the question regarding sports participation outside school time in the BEATS surveys were capturing informal activities which may explain the lack of difference between the two time points.

We did not measure opportunities for PA in the neighbourhood in this study. It is possible that the geographical context of Dunedin is internationally unique in its provision of opportunities to be active in local neighbourhoods [48]. Similarly, we did not assess the familial context, which is an important consideration in understanding supports for PA during the COVID-19 pandemic [49]. While in the current study we observed positive shifts in PA overall, there are nonetheless important opportunities to support PA maintenance during pandemics, with policy implications in the eventuality of future pandemics. Drawing from the current study and extant literature base, these could include supporting participation in organised outdoor activities while meeting physical distancing requirements, provision of online activity programmes, infrastructural shifts to facilitate safe walking and cycling around neighbourhoods, and taking a whole-of-family approach to support families to be active together.

Though our analyses noted a higher proportion of adolescents meeting ST recommendations post-COVID, fewer than one in five were doing so. Furthermore, adolescents at S2 reported lower levels of both weekday and weekend day ST than those at S1. These findings are inconsistent with those of a systematic review that reported increases in ST from before to during the initial COVID-19 lockdown among children and adolescents [50]. This review also noted that children and adolescents who faced strict lockdowns reported a sharper increase in ST than those under mild restrictions. It is possible that the restrictions experienced in Dunedin were less stringent than elsewhere. It is also possible that the pattern of ST consumption observed in our study reflects a secular trend toward lower engagement in outside school ST independent of any COVID-19 effects. However, this will require further scrutiny.

Healthy food choices and dietary patterns contribute to reducing the risk of non-communicable diseases, which are common in adulthood and are increasingly diagnosed in adolescents [51, 52]. Consumption of the recommended number of daily servings of fruits (≥ 2 servings) and vegetables (≥ 3 servings) is part of a healthy dietary pattern yet is often not achieved in all age groups, including adolescents [9, 53]. Low intakes of fruits and vegetables have an impact on health outcomes, for example, an estimated 2 million deaths worldwide were attributable to low intakes of fruit [53]. In this study, inadequate weekly intakes of fruit and vegetables was observed before and following the onset of the COVID-19 pandemic in New Zealand and there was almost no change between the two time periods. The fact that there was no significant change in fruit and vegetable intakes is interesting, as dietary patterns and food intakes can be sensitive to major national and international events [54, 55]. In contrast, the frequency of consuming soft drinks and fast foods decreased. While a reduction in the intake of sugar sweetened beverages has been observed [56], this was during periods of COVID-19 control lockdowns, whereas our observations related to periods of both lockdowns and no lockdowns but may have been influenced by recall bias or factors that our survey did not assess [55] and this may have influenced discretionary spending on non-essential food items like sugar sweetened beverages.

It is important to note that results presented in this article are based on data collected from two different samples (i.e., not repeated measures). Therefore, these results are not based on the analysis of change in the same study sample but rather on the analysis of differences between two groups of individuals of similar ages at different time points, that is, two cross-sectional surveys. Although data analysis was adjusted by age, gender, ethnicity and neighbourhood deprivation score, the findings could be attributed to inter-individual variations in lifestyle behaviours.

### Implications

In line with the World Health Organization’s Global Strategy on Diet, Physical Activity and Health [57], lifestyle behaviours such as PA, time spent being sedentary, and eating habits and their relationships to healthy weights are often targeted in combination as part of population-based strategies to promote healthy living [58]. In this study, the number of adolescents meeting guidelines for all three health behaviours considered remained very low overall, despite a small significant increase observed over time. This circumstance highlights the rationale for implementing comprehensive multi-pronged approaches to health promotion in schools, and specifically for school-based lifestyle interventions targeting multiple health behaviours, which have shown promise of effectiveness at the secondary school level [24].

### Study strengths and limitations

The strengths of this study include a large representative sample of adolescents from the study city, 100% school recruitment rate at both S1 and S2, and use of validated questions for self-reporting of PA, ST and dietary behaviours by adolescents. Study limitations include the repeated cross-sectional study design that prevents examination of individual change over time, reliance on self-report measures, which may lead to non-response and recall and social desirability biases, no assessment of duration of PA and sport participation sessions, no assessment of mobile phone time under ST, no exclusion of homework from the ST assessment, and data collection in a single city that may limit the generalizability of findings. Total screen use includes computer use for homework and as such outside school ST in the current study may not represent the actual recreational ST of participants. Future population-based studies using longitudinal designs incorporating sufficient points in time to capture relevant milestones of the COVID-19 pandemic, and a combination of self-report and device-based measures, as appropriate, will contribute to address some of the shortcomings of the present study and to provide a more complete and accurate picture of the effects of the pandemic on the behaviours of interest.

## Conclusions

Compared to pre-pandemic levels, during the first two years of the COVID-19 pandemic in New Zealand, participating adolescents self-reported higher levels of PA and consumption of sweets, and lower weekday and weekend day ST and consumption of soft drinks. Despite some observed improvements, findings highlight the need for longitudinal research combining self-reports and device-based measures, as appropriate, to develop a deeper understanding of temporal behavioural patterns of interest, which can in turn inform comprehensive health promotion for adolescents and policy preparedness and response in the advent of other pandemics.

### Supplementary Information

**Additional file 1.**

## Data Availability

Data used in data analysis for this project will not be shared due to sensitivity of the collected data as well as participants having been given assurances that the collected data will not be shared. For any queries about data used in this analysis, please contact Adjunct Professor Sandra Mandic at sandy.mandic@aut.ac.nz.

## Abbreviations

BEATS: Built Environment and Active Transport to School
MVPA: Moderate-to-vigorous physical activity
PA: Physical activity
ST: Screen time
