# Supplementary material for: Physical activity, screen time and dietary behaviours in New Zealand adolescents prior to and following the onset of the COVID-19 pandemic
Source: BMC Public Health. 2024 Jan 16;24:188. doi: 10.1186/s12889-024-17688-7 (PMC10790521; doi:10.1186/s12889-024-17688-7)
Supplement: Supplementary file 1 — Additional file 1. [file 12889_2024_17688_MOESM1_ESM.docx]

**Online supplement:
BEATS Student Survey – Mapped Questions for Analysis of Adolescents’ Health Behaviours**

| **Health behaviour** | **Survey item(s)** | **Recoding and preparation for data analysis** |  |
| --- | --- | --- | --- |
|  | | | |
| **Physical activity** | The next few questions will ask you about your physical activity habits.  **Physical activity is any activity that increases your heart rate and makes you get out of breath some of the time.**  Physical activity can be done in sports, school activities, playing with friends, or walking to school. Some examples of physical activity are running, brisk walking, cycling, roller blading, dancing, skateboarding, swimming, soccer, basketball, rugby and surfing.    For this next question, add up all the time you spent in physical activity each day.  **Over the past 7 days, on how many days were you physically active for a total of at least 60 minutes per day?**   - 0 days 🔾 1 day 🔾 2 days 🔾 3 days 🔾 4 days 🔾 5 days 🔾 6 days 🔾 7 days | These data were used to calculate and report the average number of days per week that adolescents self-reported participating in moderate-to-vigorous physical activity.  This variable was also used to categorise adolescents as meeting PA guidelines if they self-reported ≥60 minutes of MVPA for 7 days. |  |
|  |  |  |  |
| **Sport participation** | **Are you involved in any sport or do you belong to any sports teams?**   \|  \| Yes \| No \| \| --- \| --- \| --- \| \| At school? \|  \|  \| \| Outside of school? \|  \|  \| | Proportion of adolescents participating in sports at school and outside school was analysed and reported. |  |
|  |  |  |  |
| **Screen time** | The next four questions will ask about your other activities outside school including TV watching, playing computer games and internet use.  About how many hours do you usually watch **television (including streaming movies or TV shows and DVDs)** in your free time?   \|  \| None at all \| Rarely \| About half an hour a day or less \| About 1 hour a day \| About 2 hours a day \| About 3 hours a day \| About 4 hours a day \| About 5 hours a day \| About 6 hours a day \| About 7 or more hours a day \| \| --- \| --- \| --- \| --- \| --- \| --- \| --- \| --- \| --- \| --- \| --- \| \| Weekdays \|  \|  \|  \|  \|  \|  \|  \|  \|  \|  \| \| Weekend \|  \|  \|  \|  \|  \|  \|  \|  \|  \|  \|  \|  \| None at all \| Rarely \| About half an hour a day or less \| About 1 hour a day \| About 2 hours a day \| About 3 hours a day \| About 4 hours a day \| About 5 hours a day \| About 6 hours a day \| About 7 or more hours a day \| \| --- \| --- \| --- \| --- \| --- \| --- \| --- \| --- \| --- \| --- \| --- \| \| Weekdays \|  \|  \|  \|  \|  \|  \|  \|  \|  \|  \| \| Weekend \|  \|  \|  \|  \|  \|  \|  \|  \|  \|  \|   About how many hours a day do you usually **play games on a computer or games console (PlayStation, Xbox etc.)** in your free time?  About how many hours a day do you usually **use a computer for chatting on-line, internet, emailing, homework etc**. in your free time?   \|  \| None at all \| Rarely \| About half an hour a day or less \| About 1 hour a day \| About 2 hours a day \| About 3 hours a day \| About 4 hours a day \| About 5 hours a day \| About 6 hours a day \| About 7 or more hours a day \| \| --- \| --- \| --- \| --- \| --- \| --- \| --- \| --- \| --- \| --- \| --- \| \| Weekdays \|  \|  \|  \|  \|  \|  \|  \|  \|  \|  \| \| Weekend \|  \|  \|  \|  \|  \|  \|  \|  \|  \|  \| | Total weekly outside school screen time was calculated by combining weekday and weekend estimates for TV watching, playing computer games and internet use and included homework.  To calculate average screen time, collected categorical screen time data for each survey item were recoded as follows: ‘none at all’ = ‘0 hours/day’; ‘rarely’ = ‘0.1 hours/day’; ‘about half an hour a day or less’ = ‘0.5 hours/day’; ‘about 1 hour per day’ = ‘1 hour/day’; ‘about 2 hours per day’ = ‘2 hours/day’; ‘about 3 hours per day’ = ‘3 hours/day’; ‘about 4 hours per day’ = ‘4 hours/day’; ‘about 5 hours per day’ = ‘5 hours/day’; ‘about 6 hours per day’ = ‘6 hours/day’; and ‘about 7 or more hours per day’ = ‘7 hours/day’.  A threshold value of ≤2 hours of outside school screen time per day was used to identify adolescents meeting screen time guidelines |  |
| Dietary behaviours | How many times a week do you usually eat or drink...?   \|  \| Never \| Less than once a week \| Once a week \| 2-4 days a week \| 5-6 days a week \| Once a day, every day \| Every day, more than once \| \| --- \| --- \| --- \| --- \| --- \| --- \| --- \| --- \| \| Fruits \|  \|  \|  \|  \|  \|  \|  \| \| Vegetables (including frozen vegetables) \|  \|  \|  \|  \|  \|  \|  \| \| Sweets like lollies, chocolate or chocolate bars \|  \|  \|  \|  \|  \|  \|  \| \| Snack foods like chips, muesli bars or crackers \|  \|  \|  \|  \|  \|  \|  \| \| Coke, other soft drinks or energy drinks (NOT diet drinks) \|  \|  \|  \|  \|  \|  \|  \| \| Fast food or takeaway food \|  \|  \|  \|  \|  \|  \|  \| | Dietary habits data were not normally distributed. Therefore, participants’ responses were subsequently recoded into 3-category variables for weekly consumption in each food category: ‘once a week or less', '2 to 4 times per week' or '5 or more times per week' for descriptive analysis of dietary consumption patterns.  For assessing compliance with dietary recommendations, participants who self-reported consuming both fruit and vegetables “every day, more than once” were classified as meeting fruit and vegetable recommendations. |  |
